# Development of a Telemetric, Miniaturized Electrochemical Amperometric Analyzer

**DOI:** 10.3390/s17102416

**Published:** 2017-10-23

**Authors:** Jaehyo Jung, Jihoon Lee, Siho Shin, Youn Tae Kim

**Affiliations:** IT Fusion Technology Research Center, Department of IT Fusion Technology, Chosun University, 309 Pilmun-daero, Dong-gu, Gwangju 61452, Korea; jh.jung207@gmail.com (J.J.); jihoon1493@gmail.com (J.L.); shincoh1@gmail.com (S.S.)

**Keywords:** indium tin oxide (ITO), electrochemical sensor, potentiostat, amperometry, portable analyzer, wireless communication

## Abstract

In this research, we developed a portable, three-electrode electrochemical amperometric analyzer that can transmit data to a PC or a tablet via Bluetooth communication. We performed experiments using an indium tin oxide (ITO) glass electrode to confirm the performance and reliability of the analyzer. The proposed analyzer uses a current-to-voltage (I/V) converter to convert the current generated by the reduction-oxidation (redox) reaction of the buffer solution to a voltage signal. This signal is then digitized by the processor. The configuration of the power and ground of the printed circuit board (PCB) layer is divided into digital and analog parts to minimize the noise interference of each part. The proposed analyzer occupies an area of 5.9 × 3.25 cm^2^ with a current resolution of 0.4 nA. A potential of 0~2.1 V can be applied between the working and the counter electrodes. The results of this study showed the accuracy of the proposed analyzer by measuring the Ruthenium(III) chloride (${\mathrm{Ru}}^{\mathrm{III}}$) concentration in 10 mM phosphate-buffered saline (PBS) solution with a pH of 7.4. The measured data can be transmitted to a PC or a mobile such as a smartphone or a tablet PC using the included Bluetooth module. The proposed analyzer uses a 3.7 V, 120 mAh lithium polymer battery and can be operated for 60 min when fully charged, including data processing and wireless communication.

## 1. Introduction

Improvements in technology ensure continual developments in point-of-care testing (POCT) [1]. POCT, which can be used to diagnose personal health conditions by extracting a small blood sample, is widely used to managing diseases. For example, a glucose analyzer, a representative POCT device, is used to check the glucose concentration in a patient’s blood by converting the chemical reaction generated in a biochemical sensor into an electrical signal [2]. Such a device is beneficial to those who want to manage their health parameters because, in addition to having a simple design, it is also inexpensive and compact [3].

An electrochemical analysis based on amperometry that is one of the methods used to measure the current induced by a reduction-oxidation (redox) reaction at an electrode, is a suitable analysis method for the previously mentioned devices. A potential is applied between the working and the counter electrodes using a potentiostat and the current from the redox reaction is measured [4,5]. A reference electrode is used to maintain a stable potential voltage during the reaction [6,7].

i-STAT (Abbott) is one of the available electrochemical analyzers [8]. The i-STAT system uses a separate cartridge to store the blood sample, from which it then detects various biomarkers, including troponin I, B-type natriuretic peptide (BNP), and glucose, and facilitates medical professionals in gauging health conditions of patients. Another electrochemical analyzer, CHI-1040C (CH Instrument, Bee Cave, TX, USA), is a multi-potentiostat that can precisely check eight electrochemical signals simultaneously, and analyze a wide range of materials using diverse methods, such as cyclic voltammetry and differential pulse voltammetry [9]. Yet another, EmStat (PalmSens, Houten, The Netherlands), is a portable multi-channel analyzer based on three electrodes and can detect redox reactions in various analyses [10]. It includes four-channel working, counter, and reference electrodes. The above-mentioned devices based on electrochemical analysis are applied in a variety of fields, such as for lactate, toxin, and gas analyses [11,12,13].

In electrochemistry, immunosensors have been continuously researched to detect not only specific materials in solution, but also diseases in humans and animals [14,15,16]. These sensors are used to detect redox reactions at the electrode, or immune response using a potentiostat. Immune reactions are detected using antibodies labeled with enzymes, where the labeled immunosensor surface produces electroactive species that are amperometrically monitored at the electrode. The result is proportional to the amount of antibody targets anchored to the surface of the sensor. The electrochemical analyzer detects the abovementioned reaction and converts it into digital data such that the results can be visualized [17]. Hence, they can recognize targets in samples like blood and can, therefore, be applied to POCT for medical diagnoses [18,19]. However, conventional instruments are too expensive, and their use for study or medical diagnosis requires the skill of an expert [20,21]. Moreover, existing devices are cumbersome to use and require complex procedures and an established setting to measure a solution. Common portable devices can be used for various purposes to analyze materials, but they are expensive and it is difficult to detect specific targets such as diseases for personal or family use [22]. Further, commercial portable analyzers are smaller than usual systems but are not suitable for application to other devices such as wearables and point of care systems for the public.

Early research employed custom connectors or cables to connect the sensor to the analyzer to measure electrochemical signals, i.e., the sensors required specific instruments in experiments [23]. Furthermore, commercial chips or devices were often used, but this made it difficult to modify circuits and software for applications in other fields, and to transfer the data to a PC via wired means, making these instruments less suitable for portable use [24,25]. To conduct biochemical experiments, researchers need additional instruments, such as zigs, to use sensors of suitable size and structure.

In this study, we have developed a miniaturized amperometric analyzer that can be applied for POCT, or as a portable system. We used micropatterned ITO glass as the electrode to measure the electrochemical activity. For user convenience, the proposed analyzer was designed to communicate with a smartphone, a tablet PC, or a desktop using Bluetooth. A user can remotely control the analyzer and confirm the result data using a PC or mobile with wireless communication. This analyzer is based on the amperometry method.

## 2. Materials and Methods

### 2.1. Design of Amperometric Device

**Figure 1:**
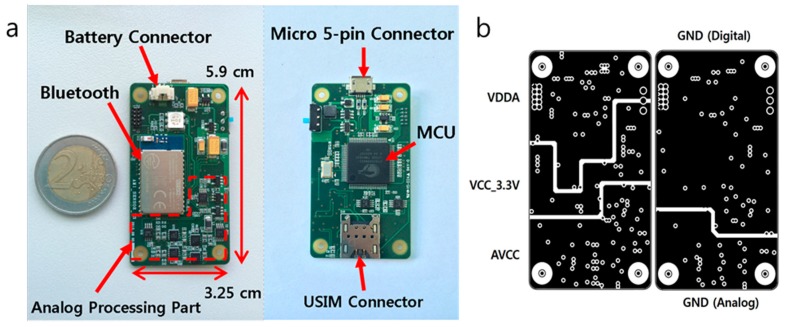
(**a**) PCB of miniaturized amperometric analyzer; (**b**) Scheme of the ground and power layer.

Figure 1 shows the printed circuit board (PCB) configuration of the proposed analyzer. It comprises a data processor that includes a microcontroller unit (MCU), along with analog processing, power, and communication parts. To accurately measure the microcurrent generated by an electrochemical reaction, a universal subscriber identity module (USIM) connector, a connector that is connected to a piece of ITO glass, is placed near the components used for analog processing. The use of the shortest possible distance for the circuit line minimizes the inflow of external noise. Because noise from the digital processing significantly affects analog processing, we alleviated the interference between the analog and digital components by dividing the power and ground layer in the PCB, as shown in Figure 1b. This structure provides a stable output signal in the tests. The electrochemical experiment uses a low potential and current; thus, very low noise has a negative impact on the result.

This division enables the accurate detection of a signal without effects from each part. In this study, we used a 3.7 V, 120 mAh lithium polymer battery for performing operations for 60 min, including data processing and Bluetooth communication. This enabled the analyzer to be operated 60 times when the measurement time was set at 50 s. The battery can be recharged through a micro 5-pin USB cable. To transmit the data processed by the MCU, a Bluetooth 4.0 module (WT-12, Bluegiga, Espoo, Finland) was used. The CY8C5888-LP based on the Cortex-M3 (Cypress, San Jose CA, USA) was used as the MCU, exhibiting a high performance and low power consumption. The CY8C5888-LP is a programmable system-on-chip (PSoC) series, the use of which made it possible to generate an analog block in the MCU via programming. This enables 12-bit digital-to-analog conversion (DAC) in the MCU and easy editing of the device functions. The PCB occupies an area of 3.25 × 5.9 cm^2^. Table 1 shows the performance of the proposed system.

**Figure 2:**
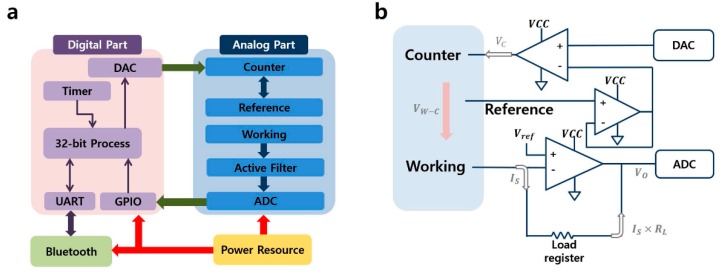
(**a**) Device block diagram; (**b**) Configuration of sensing part.

The power source of the analyzer supplies power to various functions including Bluetooth, the digital part, and the analog part, as shown in Figure 2a. The analog part consists of a potentiostat, including the counter, reference, and working circuits, an active filter, and a 16-bit analog-to-digital converter (ADC). An OPA2376 (Texas Instruments, Dallas, TX, USA) op-amp is used for the reference, counter, and active filter circuits. An OPA381 (Texas Instruments), a high-resolution current-to-voltage (I/V) converter is used for the working circuit. When a potential is applied to a solution, the reference circuit stabilizes the output voltage of the counter circuit using a feedback loop.

The electrochemical signal from the analog part is converted into a digital value via the ADC and is then transmitted to the MCU. The data is processed into packets for wireless communication. Figure 2b shows the current flow generated by the redox reaction. V_ref_, which is the basis for the working voltage V_W_, has a value of 1.2 V, and V_C_ is controlled by the DAC. Potential V_P_ is the differential voltage between the working and the counter electrodes (V_P_ = V_W_ − V_C_), and R_S_ is the solution or sample resistance. The electrochemical reaction of the solution represented by the potential produces I_S_, which is equal to V_P_ ÷ R_S_, based on Ohm’s law. This current flows in the load register R_L_, which has a value of 1 MΩ, and is then converted to the load voltage V_L_ = I_S_ × R_L_.

Finally, a readable voltage V_O_ is produced by the sum of V_W_ and V_L_ before being input to the ADC. We performed an experiment with a sample resistance of 10 MΩ and a potential of 500 mV to assess the performance of the analyzer. The values of V_W_ and V_L_ were 1200 and 50 mV, respectively, indicating that a current of 1 nA is equivalent to a potential of 1 mV.

The proposed analyzer device adopts Bluetooth communication. A manager device such as a PC, smartphone, or tablet PC connected using Bluetooth provides the analyzer with settings for the potential, maximum number of samples, and sampling interval. Electrochemical analysis starts immediately after the setting information established by the manager is transferred and continues for the entire runtime, which is the count number multiplied by the interval. When the operation is completed, the result data are transmitted to the manager, where it can be represented graphically and stored in a text file format.

### 2.2. System Operation Flow

**Figure 3:**
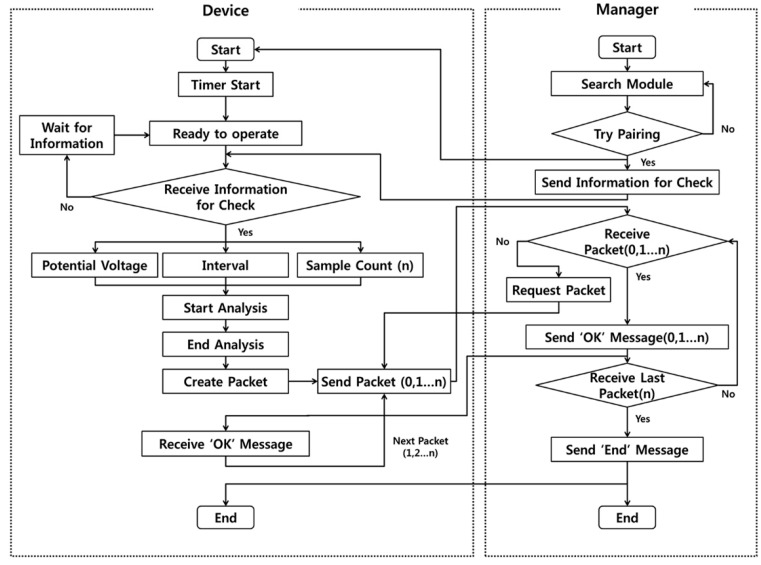
System operation flow via Bluetooth communication.

The data process between the analyzer and the manager is shown in Figure 3. When the Bluetooth pairing between the analyzer and the manager is successful, the analyzer is ready to activate the analysis and start the timer. When the analyzer receives information regarding the requirements for a run, it starts analyzing the solution during the allocated time. All the results are converted into packets for Bluetooth communication after the completion of the measurement. The packets are numbered and transmitted to the manager in a numerical order through the Bluetooth module. Typically, when a packet arrives at the manager, the manager sends the analyzer an acknowledgement message (ACK) as per the sequence. When the analyzer receives an ACK, the next packet is transferred. Using these steps, when the last packet is transmitted, the entire operation is completed after the manager sends a termination message to the analyzer.

### 2.3. ITO Glass Sensor

In this study, an electrochemical electrodes sensor manufactured by micropatterned ITO glass was used for signal analysis. This sensor consisted of working, reference, and counter electrodes. The reference and counter electrodes treated with silver (Ag) and silver-silver chloride (Ag/AgCl) paste, respectively, in order to optimize the detection of an electrochemical signal. The use of ITO glass for electrochemical electrodes offers numerous advantages, such as a wide potential window, low capacitive currents, and highly practical fabrication of electrodes [26].

**Figure 4:**
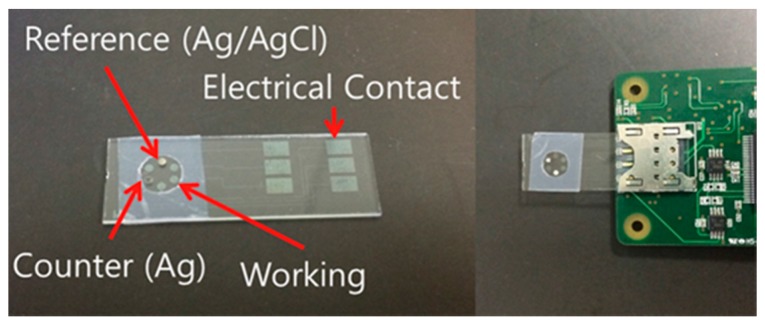
Patterned electrodes fabricated using indium tin oxide (ITO) glass.

The Pattern of an ITO sensor fabricated using the same specifications as those of the USIM connector is shown in Figure 4. In this study, the unmarked electrodes were not used, and the wire-connected sensor was disconnected in the test because the remainder of the ITO electrode could adversely influence results. The unnecessary area of the face was blocked except for the electrode face that is in direct contact with the solution.

## 3. Results and Discussion

We established settings for the experiment after connecting the Bluetooth dongle to a PC and enabling the pairing with the analyzer. Each experiment used 10 µL of the solution, at the ITO sensor. The experiment was performed at a potential of 0.2 V with a measurement time of 50 s. The measurement result was displayed in terms of the current and an integrated charge with respect to time.

**Figure 5:**
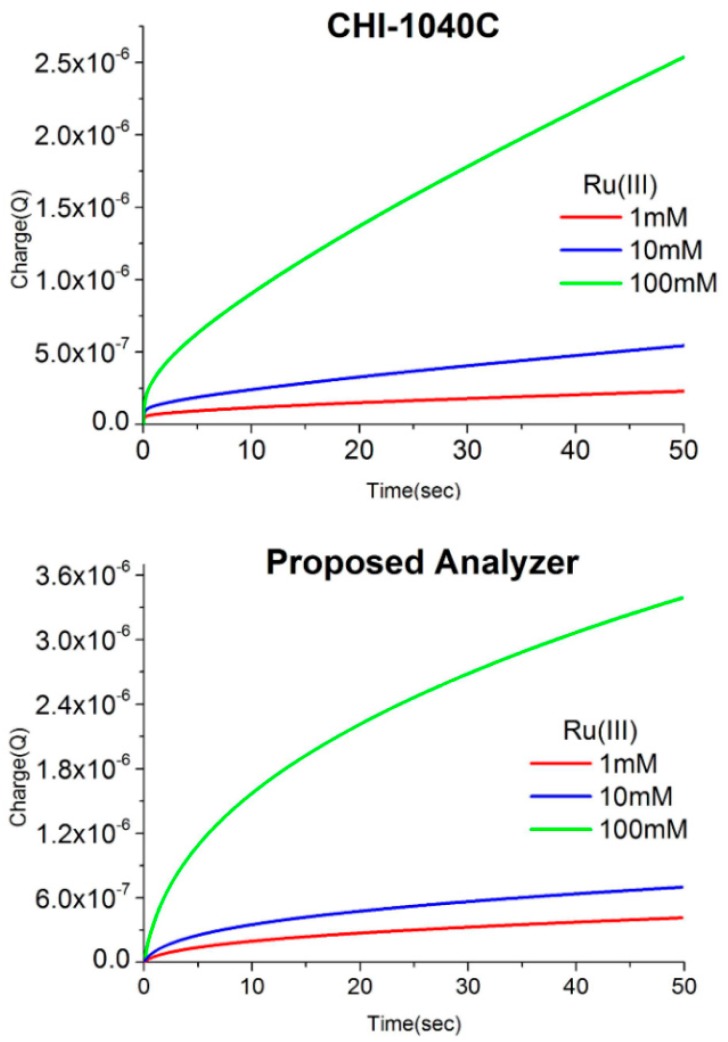
Charge variation with proposed analyzer and CHI-1040C.

For performance verification, we compared the proposed analyzer with CHI-1040C using the following solutions: 1 mM, 10 mM, and 100 mM of Ruthenium (III) chloride (${\mathrm{Ru}}^{\mathrm{III}}$) in 10 mM phosphate-buffered saline (PBS) at 7.4 pH. Figure 5 shows the graphs of the research results. The results of experiments with the proposed analyzer and CHI-1040C are presented for various solution concentrations to confirm the redox reaction. Although the resulting data sets have slight discrepancies between them, we could still identify a linear increase of the signal by increasing the concentration tenfold.

**Figure 6:**
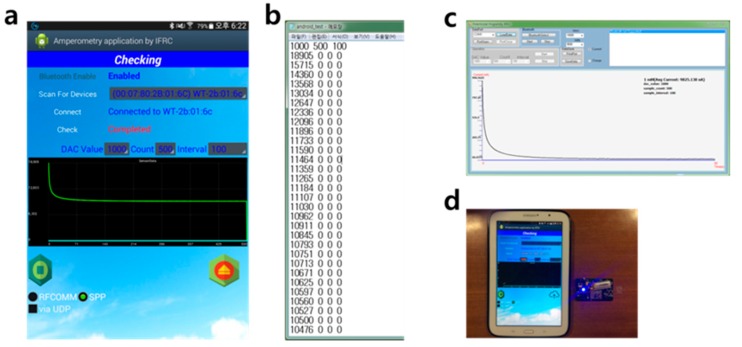
(**a**) Results for 1 mM ${\mathrm{Ru}}^{\mathrm{III}}$ shown on smart tablet; (**b**) Result data in text file format; (**c**) Graph of the current in PC program; (**d**) Smart table used in experiment.

Figure 6a shows the measurement results for 10 mM of ${\mathrm{Ru}}^{\mathrm{III}}$ in an environment identical to the previous one and displayed using a tablet PC. This experiment is operated by application program. When the test is finished, the results are saved in the form of a text file, as shown in Figure 6b. Here, the voltage values are digitized by ADC. This data is converted to a current value, which can be displayed using a PC, through a simple modification, as shown in Figure 6c, after being saved to a web server via mobile communication (Wifi, 3G, or 4G). In this study, a Samsung tablet (SHW-M500W, Seoul, Korea) equipped with a Bluetooth 4.0 module is used and an application was developed based on an Android 4.1 (Jelly Bean) and web server was configured by APMSETUP 7 for Win-32. The PC program was developed using Visual Studio 2010, C# based on Windows 7 32-bit.

We have developed an accurate and an inexpensive electrochemical analyzer based on amperometry. The proposed analyzer can communicate with various other devices through Bluetooth communication and has an intuitive interface that is user-friendly. However, the capacity of packet storage in the MCU can be exceeded by an increase in the sample amount. Real-time processing communication, wherein a packet of processing data is transmitted to terminal immediately, without waiting construction of whole packet, is appropriate to alleviate this situation. Therefore, it is necessary to synchronize the data-processing rate and the Bluetooth communication rate, when data is transmitted immediately after generating each packet. If both rates are different, the system will operate abnormally or stop. To solve this problem, an additional study is needed about the synchronization of processing and Bluetooth transmitting rates. From the results of this research, we expect a faster data process, as well as low battery consumption by reduction of operating time from analysis to termination of communication.

In the main study, the developed analyzer is operated on the basis of Bluetooth communication. Bluetooth communication based on version 4.0 takes approximately 45 ms to transmit a data packet and receive a confirmation response at a baud rate of 115,200 bps. The amount of time spent in transmitting all of the data increases as the number of samples increases. If an abnormal packet is sent to the analyzer during communication, measurement is interrupted. To prevent the cessation of all operations in the event of lost or damaged packets during the communication process, the analyzer was designed to retransfer these packets from the device to the manager using a stop-and-wait protocol. We secured the operational stability of the proposed analyzer using this design. Subsequently, it was possible to apply the proposed analyzer to the ISO/IEEE 11073 standard protocol because the WT-12 attached to the PCB supports a personal health device (PHD) with Bluetooth 4.0. By developing a wireless healthcare based on the researched analyzer with the application of this protocol, we expect to achieve enhance data transmission efficiency and compatibility with other communication devices [27].

An ITO glass sensor with the pattern shown in Figure 4 was used to measure a single signal. An optimal signal was detected in a processing experiment with identical settings for the distances of the counter, reference, and working electrodes. However, additional research is required to change the magnitude of the electrode and the pattern to eliminate the oxygen-bubble phenomenon. This can be caused by a redox reaction, depending on multichannel signal detection, and the solution or experimental circumstances [28,29]. Research on the development of an electrochemical immunosensor that can detect specific biomarkers by applying an antibody fixation technology to ITO is currently underway [30]. Such a sensor is expected to be applied to a new field of POCT, as well as U-healthcare. This application will enable the diagnosis of diseases, such as through the use of cardiac markers, by combining this type of sensor with the analyzer developed in this study.

## 4. Conclusions

In this study, we developed a portable three-electrode electrochemical analyzer that measures the current generated using an electrochemical sensor based on amperometry and wirelessly communicates the result via Bluetooth. This electrochemical analyzer was developed by our research. The size and weight of the proposed analyzer is 22.5% and 30% lower than commercial reference systems, respectively. Furthermore, the proposed analyzer was designed such that the potential and measurement time can be set through a convenient interface, which is used to save and display the results graphically, as well as numerically. Because the PSoC MCU, CY8C5888, supports programmable analog functions, the functions can be actively changed or developed based on their usage, without changing the circuits. The MCU and Bluetooth modules both consume low power and exhibit high performance. The system has no difficulty operating with a low-capacity battery (120 mAh), which can be replaced with a battery of higher capacity. We measured samples using micropatterned ITO glass and identified the differences in the concentrations of the solutions in the experiments. Thus, we proved the possibility of performing electrochemical analysis using a cheaply produced device. We expect that it will be applied to wearable devices for the early detection of various diseases found in daily life.

## Figures and Tables

**Table 1:** System specification.

Specification	
Electrode	ITO glass (CE: Ag, RE: Ag/AgCl)
Technique	Chronoamperometry, Chronocoulometry
Channel	1–4 ch
Potential range	±1.1 V
Potential step	10 mV
Sampling interval	10–1000 msec
Sampling count	100–3000
Current resolution	0.4 nA
Size	3.25 × 5.9 × 0.9 cm
Weight	35 g
Power supply	Li-polyer 120 mAh
Display	PC, Tablet PC
